# Pros and Cons of Dual-Energy CT Systems: “One Does Not Fit All”

**DOI:** 10.3390/tomography9010017

**Published:** 2023-01-27

**Authors:** Ana P. Borges, Célia Antunes, Luís Curvo-Semedo

**Affiliations:** 1 Medical Imaging Department, Coimbra University Hospitals, 3004-561 Coimbra, Portugal; 2 Faculty of Medicine, University of Coimbra, 3000-370 Coimbra, Portugal; 3 Academic and Clinical Centre of Coimbra, 3000-370 Coimbra, Portugal

**Keywords:** dual-energy CT, spectral CT, dual-source CT, fast kVp switching, dual-layer detector CT, split-filter, image quality, photon counting

## Abstract

Dual-energy computed tomography (DECT) uses different energy spectrum x-ray beams for differentiating materials with similar attenuation at a certain energy. Compared with single-energy CT, it provides images with better diagnostic performance and a potential reduction of contrast agent and radiation doses. There are different commercially available DECT technologies, with machines that may display two x-ray sources and two detectors, a single source capable of fast switching between two energy levels, a specialized detector capable of acquiring high- and low-energy data sets, and a filter splitting the beam into high- and low-energy beams at the output. Sequential acquisition at different tube voltages is an alternative approach. This narrative review describes the DECT technique using a Q&A format and visual representations. Physical concepts, parameters influencing image quality, postprocessing methods, applicability in daily routine workflow, and radiation considerations are discussed. Differences between scanners are described, regarding design, image quality variabilities, and their advantages and limitations. Additionally, current clinical applications are listed, and future perspectives for spectral CT imaging are addressed. Acknowledging the strengths and weaknesses of different DECT scanners is important, as these could be adapted to each patient, clinical scenario, and financial capability. This technology is undoubtedly valuable and will certainly keep improving.

## 1. Introduction

Dual-energy computed tomography (DECT) is a computed tomography (CT) technique that uses different energy x-ray beams to differentiate materials with the same attenuation at a certain energy, and which are therefore not distinguishable with single-energy CT (SECT) [1,2,3,4]. This imaging technique, which is being increasingly implemented in daily clinical practice, offers improved diagnostic performance over SECT, the possibility of depicting and quantifying specific materials, and potential reduction of contrast agent and radiation doses. However, several DECT systems are currently being used in the clinical routine that are technically different and have individual advantages and limitations which are important to know [5,6,7,8]. In this way, adaptation of the technique to each patient, clinical scenario, and financial capability can be made easier. Therefore, in this review, we address the different DECT systems, with an emphasis on their own strengths and weaknesses.

## 2. Materials and Methods

**Figure 1:**
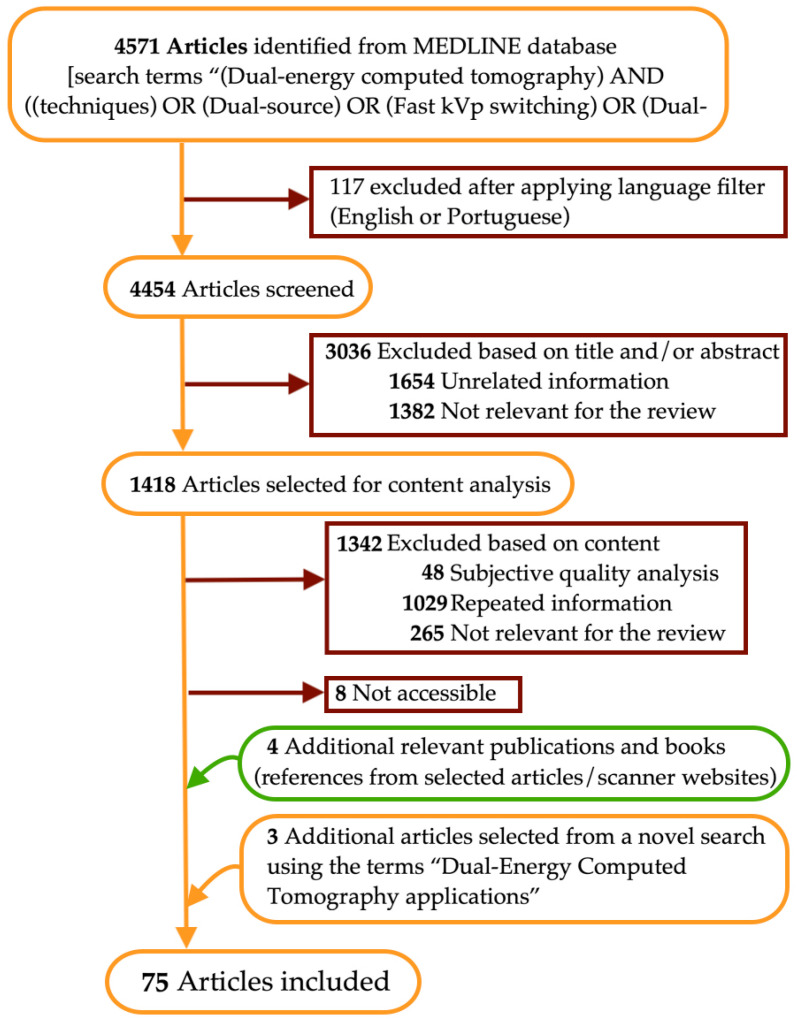
Flow diagram of the narrative review of the literature.

We conducted a narrative review of the literature on DECT technique. To that end, we searched the MEDLINE database for papers published from January 2010 to October 2022, using the search terms “(Dual-energy computed tomography) AND ((techniques) OR (Dual-source) OR (Fast kVp switching) OR (Dual-layer detector) OR (Split-filter) OR (Twinbeam))”. The flow diagram of the narrative review of the literature is presented in Figure 1.

Out of 4571 articles, 112 written in languages other than English or Portuguese were excluded. Then, the most relevant articles related to the search terms were chosen by means of titles and abstracts analysis, as well as article content when justified. Specifically, we reviewed studies describing DECT technique and comparing different features of currently available DECT systems, namely in terms of design, strengths, and limitations. Articles were excluded based upon a subjective quality analysis of structure, as well as the presence of repeated information obtained from more relevant records, unrelated topics, and information considered not relevant for the review. Additional relevant publications and books were identified in the references cited in the selected papers. Information sections on each DECT platform website were also assessed. In addition, we reviewed the most used current clinical applications of DECT (information obtained from the first search and a novel search using the terms “Dual-Energy Computed Tomography applications”, 758 results), and the future perspectives for spectral CT imaging (information obtained from the first search). The included studies are listed in Table 1, with indications of the topics of analysis for the review.

All the relevant information gathered was structured in a question-and-answer format, and visual representations based on the reviewed information were drawn using Microsoft Corporation software.

## 3. Results

### 3.1. What is DECT and What Does it Add to Single-Energy CT?

Differentiating tissues on CT images is made more difficult by the possible overlap in their linear attenuation coefficient (μ) at a given energy of x-rays, which depends on material composition, mass density, and interacting photon energies [1]. Scanning with a different energy spectrum x-ray beam, DECT allows the differentiation of materials with the same attenuation at certain energy (such as iodine and calcium at 100 keV) but different attenuation curves (μ as a function of energy) [1,2,3].

The process is far from new: it was initially described in 1973 using two acquisitions of different energies, and was further investigated in 1976, when it was shown possible to differentiate attenuation coefficients even with polyenergetic x-ray spectra [1]. Since then, technological advances allowed the development of several DECT systems, with clinical use introduced in 2006 and further continued refinement [5].

Advantages of DECT over SECT include greater diagnostic performance due to higher iodine contrast-to-noise ratio (CNR), reduced beam-hardening artifacts, and the possibility of material-specific images. Additionally, it requires lower concentrations of contrast agents and allows radiation dose reduction by its capability of generating virtual non-contrast (VNC) images [6,8,9].

### 3.2. What Physical Concepts Are Important to Understand?

**Figure 2:**
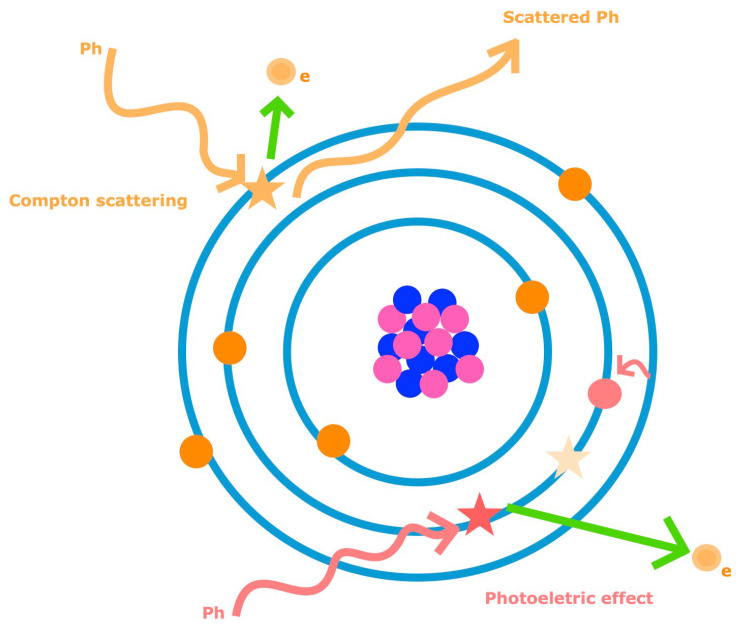
Schematic representation of the photoelectric effect and Compton scattering. The photoelectric effect is the ejection of an inner K-shell electron (e) of an atom consequent to the interaction of an incident photon (Ph), with subsequent filling of the void by an adjacent shell electron (curved arrow). Compton scattering is the ejection of an outer shell electron (e) of an atom by an incident photon (Ph), resulting in photon scattering with some energy reduction.

The CT image results from the attenuation of the x-ray beam by the encountered materials, which is mainly dependent on two physical interactions. The ejection of an inner K-shell electron of an atom consequent to the interaction of an incident photon is termed photoelectric effect [10]. It requires a minimum energy (K-shell binding energy), different for each material (increasing with greater atomic numbers), above which the attenuation peaks (element K-edge). Subsequently, an adjacent shell electron fills the void (Figure 2) [11,12]. This effect predominates at lower energies and strongly depends on the atomic number of tissue elements (proportional to its cube) and photon energy (inversely proportional) [4,5,6].

The other important interaction is the Compton scattering, which consists of the ejection of an outer shell electron of an atom by an incident photon, resulting in photon scattering, with some energy reduction (Figure 1) [10]. It predominates at higher energies and depends on material electron density (dominant factor), tube voltage, and energy spectrum [5,6].

For adequate differentiation of materials, their atomic numbers must be high and differ sufficiently. Elements with low similar numbers have low and similar attenuation and cannot be distinguished, whereas those with high and largely different numbers are better depicted and differentiated [5]. Thus, heavy atoms such as calcium, iodine, barium, and xenon can be adequately differentiated from body materials with a weak photoelectric effect [6].

### 3.3. Does “Dual-Energy” Mean Two Photon Energies?

No. It refers to the use of two polychromatic photon spectra (hence the synonymous term “spectral CT”), whose maximum energy is defined by the tube voltage [13].

Typically, spectra at 80 and 140 kilovolt peak (kVp) are used, given that energies below 80 kVp generate few photons and body absorption is higher, and energies above 140 kVp (or 150 kVp for some scanners) increase the dose and reduce soft tissue contrast. In some dual-source models, the low-energy value may be higher (90/100 kVp), especially in larger patients (as it increases the total output of the low-energy spectrum and decreases the image noise), or lower (70 kVp), for pediatrics [5,10].

Filters may be used to eliminate the low-energy quanta from the high-energy spectrum, reducing the overlap with the low-energy spectrum [13].

### 3.4. Which Parameters Influence Imaging Quality in DECT?

**Figure 3:**
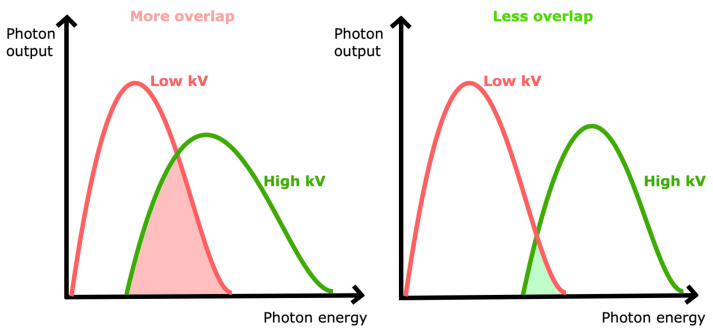
Schematic representation of different overlap between low- and high-energy x-ray spectrum. Less overlap (i.e., greater spectral separation) allows better imaging quality.

Spectral separation is important for reliable material decomposition and iodine quantification accuracy; being better with less overlap of the detected low- and high-energy x-ray spectrum means less dose is required to obtain the same image quality (Figure 3) [14].

Spatial resolution is relevant for the visualization of small objects (image “sharpness”). It has a significant impact on image noise and depends on focal spot size, detector element size, and the number of projections per rotation [12,14].

Regarding time, three parameters are important in DECT. The total scan time is important for breath hold and good contrast opacification. Temporal resolution is the ability to resolve mobile structures, defined by time for collecting full spectral data for a given image voxel or slice (about half a rotation in most scanners, therefore influenced by gantry speed). It relates to motion artifacts, which can be improved by motion compensation algorithms. Lastly, spectral projection delay is the delay between the high- and low-energy measurements, being important for material decomposition and reconstruction algorithms in the projection domain and only possible when the low- and high-energy data are aligned temporally and spatially. It relates to errors and artifacts caused by the motion between the high- and low-energy measurement, being inherent to the scanner design [12,15].

### 3.5. How Is Postprocessing Performed in DECT Imaging and Why Is It Useful?

**Figure 4:**
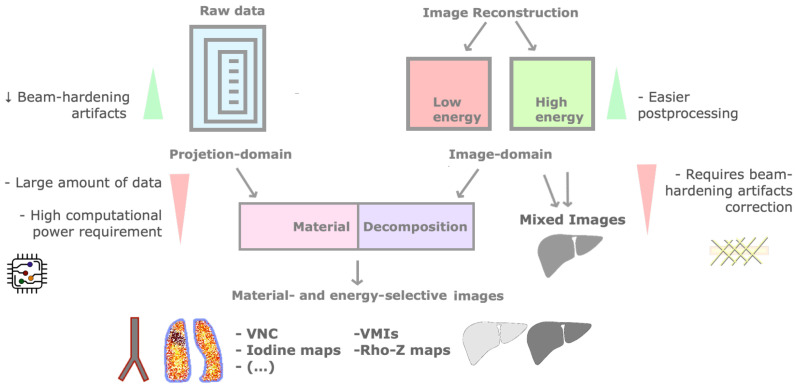
Postprocessing in DECT imaging. Postprocessing may be performed before (raw data or projection-space domain) or after (image-space domain) the reconstruction of high- or low-energy images. The former has reduced beam-hardening artifacts but requires high computational power. Material decomposition algorithms generate material- and energy-selective images. Iodine may be subtracted from material-specific images, generating a virtual non-contrast image (VNC), or color-coded, creating iodine maps. Virtual monochromatic images (VMIs) mimic single-energy scans. Mixed images are mostly used for routine diagnostic interpretation.

Postprocessing of dual-energy data may be performed before (projection-space domain) or after (image-space domain) the reconstruction of high- or low-energy images, depending on the scanner (Figure 4) [3,16]. The former has the advantage of reduced beam hardening artifacts but requires a high computational power. Postprocessing on the reconstructed images could be easier, but beam hardening artifacts need to be corrected [2,3].

Material-selective information is extracted by material decomposition algorithms, which decompose unknown tissues into selected materials, based on their attenuation plot at different energy levels [12]. They include two-material (applied to raw data) and three-material decomposition (applied to the image-space domain, respecting the conservation of mass) [2,3,10,16].

Iodine may be subtracted from material-specific images, generating VNC images [3,16]. These have shown image quality comparable to true non-contrast images, potentially obviating a true unenhanced acquisition (therefore reducing radiation exposure and scan time) [10,18,19]. However, attenuation values may be higher (especially with fat) and non-reproductible among scanners, and the size of calcifications tends to be underestimated (smaller ones may be overlooked) [20,21,22,23,24,25,26,27]. Additionally, incomplete removal of iodine may result in false positive findings [6,28]. It is possible to similarly suppress other tissues such as bone or calcium (useful in angiographic studies and bone marrow evaluation) [3,6,12]. Iodine may be color-coded, generating iodine overlay images for quantification [10,16].

Energy-selective DECT applications include Rho-Z maps (effective atomic number and electron density maps based on a semiquantitative assessment of materials) and virtual monochromatic images (VMIs) [2,3,17], which mimic a scan obtained at a single energy [8], described in terms of kiloelectronvolt (keV) instead of kVp (from 40 to 200 keV, customized to specific clinical applications) [10,12,29]. They allow the reduction of proton-starving and beam hardening artifacts and optimize the image quality [2,10,12]. Low-keV images improve contrast enhancement due to higher beam attenuation by iodine, at the cost of greater noise (reduced with noise reduction techniques and specific algorithms) [2,6,16]. High-keV images have less noise but provide less contrast and are susceptible to photon-starvation and metal artifacts [2,10,16,30]. Those with intermediate energy (60–75 keV) have balanced contrast and image noise, being ideal for evaluation of soft tissues [29]. Novel frequency split techniques combine high-contrast from low-keV images with lower noise at higher keV images [28].

Mixed or blended CT images (a weighted average of low- and high-energy images to simulate the standard 120 kVp dataset) are mostly used for routine diagnostic interpretation, without any material decomposition performed [2,3,29].

### 3.6. What Are the Technical Differences between Available DECT Systems?

**Figure 5:**
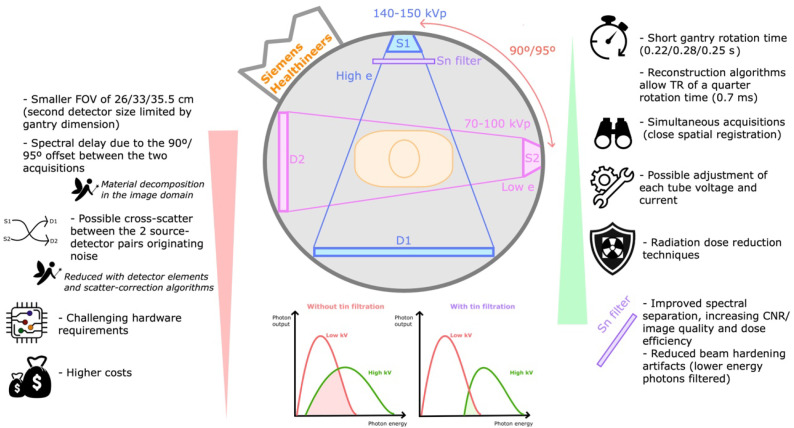
Dual-source DECT. Schematic representation of dual-source DECT scanner and its drawbacks (listed on the left) and strengths (listed on the right). These scanners (commercialized by Siemens Healthineers) are composed of two source-detector pairs dispersed almost perpendicularly. Simultaneous scans are obtained with high- and low-energy spectra (typically 80–100 kVp and 140–150 kVp). The added metallic tin (Sn) filter in the high-energy x-ray tube improves spectral separation, increasing the dose efficiency and CNR (contrast-to-noise ratio), and reduces beam hardening effects. D1—first detector 1, D2—second detector 2, e—energy, FOV—field of view, kVp—peak kilovoltage, ms—miliseconds, s—seconds, S1—first source 1, S2—second source, TR—time resolution.

The main technical differences between commercially available DECT technologies are schematically represented in Figure 5. In different ways, DECT systems acquire separate records of high- and low-energy measurements. Commercially available scanners may have two x-ray sources and two detectors, or a single source and detector [5].

Dual-source DECT (dsDECT) scanners were the first technology introduced in clinical routine in 2006, consisting of two independent x-ray tubes coupled with two independent detectors, each set mounted within the gantry with an offset of 90 or 95°, which allows acceptable temporal resolution [2,5,10]. 

Images are reconstructed from simultaneous scans obtained with each tube-detector pair at high- and low-energy spectra, allowing relatively close spatial registration [1,5,10]. The energy used is typically 80–100 kVp and 140–150 kVp depending on the model, but other combinations may be used for specific applications [5]. The second and third generation scanners add a metallic tin (Sn) filter in the high-energy x-ray tube, increasing the dose efficiency and CNR. Other advantages include the possible tube voltage adjustments to maximize spectral contrast and radiation dose efficiency, and the dose reduction technologies (e.g., automated tube current modulation, iterative reconstruction methods) [5,10,31].

Disadvantages include the smaller usable field of view (FOV) of 26, 33, or 35.5 cm, as the second detector size is limited by CT gantry dimension (although the larger detector data outside this field is still available for single-energy imaging). Possible cross-scatter between the two source-detector combinations originates bias and noise (technically lessened using detector elements and corrected with imaging reconstruction methods) [1,6]. The angle difference between the tubes results in time delays of at least 70 milliseconds (one-quarter rotation time) between each high and low projection measurements, hindering projection-based material decomposition. Another relevant drawback is the significant additional hardware requirement, increasing technical challenges and costs [5].

**Figure 6:**
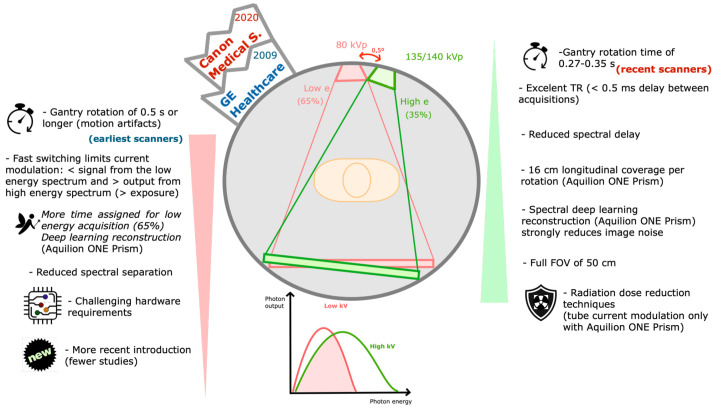
Single-source DECT with fast kVp switching of tube potential. Schematic representation of a fast kVp switching DECT scanner and its drawbacks (listed on the left) and strengths (listed on the right). These scanners (commercialized by GE Healthcare and Canon Medical Systems) are composed of a specialized generator capable of very fast switching between low- and high-energy spectra projections, which are collected separately by a specialized detector capable of fast sampling. Alternating acquisitions at 80 and 135 or 140 kVp are obtained on each rotation, with a small offset (<0.5°). Aquilion ONE Prism (Canon) offers 16 cm of longitudinal coverage and spectral deep learning reconstruction. e—energy, FOV—field of view, kVp—peak kilovoltage, s—seconds, TR—time resolution.

Single-source DECT with fast kVp switching of tube potential (Figure 6)

The first commercialized DECT scanner was the Siemens Somatom DRH in 1987, using a fast kV switching technique [14]. It was mainly used for bone mineral density analysis, but the lack of dose modulation resulted in dose penalties, so it was abandoned. The technique was re-introduced in 2010 by GE Healthcare (DiscoveryTM CT750 HD) and further refined in 2017 (RevolutionTM CT). More recently (2020), a new platform was introduced by Canon Medical Systems (Aquilion One Prism), combining deep learning-based reconstruction [14,32,33]. 

This scanner contains a specialized generator capable of very fast switching between low- and high-energy projections, which are collected separately by a detector capable of fast sampling [5,6]. Alternating acquisitions at 80 and 135/140 kVp are obtained on each rotation, with a small offset (<0.5°) [2,5]. The tube current is fixed, but the exposure time differs for each voltage (roughly 65% for low-energy and 35% for high-energy) [34].

Advantages of this technique include few delays between low and high projections (<0.5 ms), providing excellent temporal resolution, cost-efficient design and a FOV of 50 cm [2,5,34]. Additionally, material decomposition is performed in the projection domain [5,12]. Offering 16 cm of longitudinal coverage (320 detector rows), the novel Aquilion One Prism allows whole organ acquisition in a single rotation, reducing motion artifacts. In addition, its spectral deep learning reconstruction substantially reduces the image noise [33].

Limitations in current modulation during the switching lead to a relative reduction of signal from the low-energy spectrum and increased output from the high-energy spectrum. This is partly compensated for by assigning more time for the low kVp acquisition and by spectral deep learning reconstruction [1,5,33]. Other disadvantages include the gantry rotation speed of ≥0.5 s with the earliest scanners (reduced to 0.27–0.35 s with recent scanners), and the lower spectral separation [5,6,13]. Additionally, specialized hardware is required [31]. Tube current modulation for radiation dose reduction is only available with the Aquilion One Prism platform [33].

**Figure 7:**
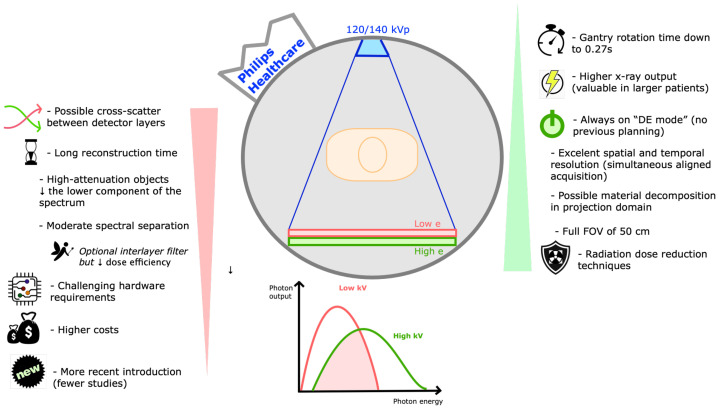
Dual-layer detector DECT. Schematic representation of a dual-layer detector DECT scanner and its drawbacks (listed on the left) and strengths (listed on the right). These scanners (commercialized by Philips Healthcare) are composed of a single source (fixed energy of 120 or 140 kVp) and a single detector with 2 layers (lower-energy photons preferably absorbed by the top layer and the bottom layer absorbs the remaining higher-energy photons). DE—dual-energy, e—energy, FOV—field of view, kVp—peak kilovoltage, s—seconds.

Dual-Layer detector DECT (Figure 7)

Dual-layer detector DECT scanners were introduced by Philips Healthcare in 2005 [2]. A single scan is obtained at a fixed high-energy (120 or 140 kVp), with spectral separation at the level of a highly specialized detector, composed of two layers with different energy sensitivity (lower-energy photons preferably absorbed by the top layer and the bottom layer absorbs the remaining higher-energy photons), and an optional interlayer filter [5]. Different detector layer thicknesses (which can be tailored) allow comparable noise in the low- and high-energy images [1,5,10].

Advantages of this scanner include the absence of delay between the two energy acquisitions, providing excellent temporal resolution, allied to an excellent spatial resolution. The constantly high kVp allows higher total x-ray power, valuable in larger patients, and the absorption of most low-energy photons by the top layer hardens the spectrum. Scanning can be performed at a FOV of 50 cm without limitation in rotation speed. The perfect alignment of acquired spectral data allows material decomposition in the projection domain, and iterative reconstructions can be used for dose reduction [2,5,12]. Finally, it is always operating in a “dual-energy mode”, so it does not require previous planning, being valuable in the workflow, at the cost of a relatively long reconstruction time [1,6]. 

Disadvantages include an unsharp distinction between lower- and higher-energy photons by the detector (overlap between the sensitivity profiles of its two layers), leading to lower energy separation (improved by the interlayer filter, although reducing the dose efficiency) [5,6]. Another challenge is that high-attenuation objects reduce the lower energy component of the spectrum. In addition, emitted low- and high-energy spectra cannot be balanced at the source; there is susceptibility to cross-scattering between the two detector layers and the use of an anti-scatter grid reduces the optical sensitivity. Finally, specialized detector hardware requirements increase technical challenges and costs, and their relatively recent introduction means there are fewer studies on clinical efficiency [2,5].

**Figure 8:**
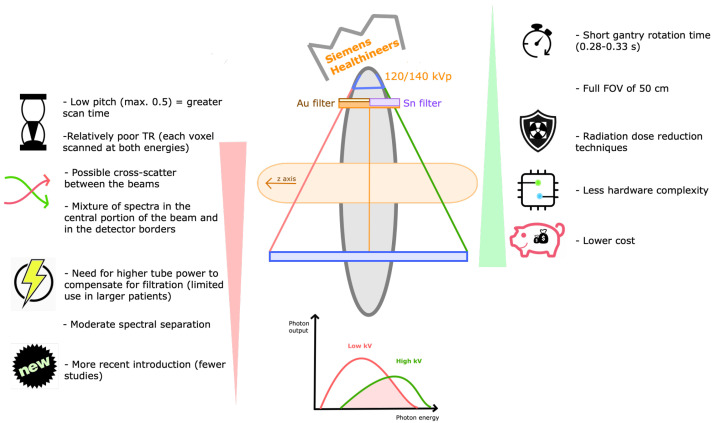
Single-source split-filter DECT. Schematic representation of single-source split-filter DECT scanner and its drawbacks (listed on the left) and strengths (listed on the right). These scanners (commercialized by Siemens Healthineers) are composed of a single source and detector, with a split-filter of gold (Au) and tin (Sn) at the x-ray tube, respectively, filtering the low- and high-energy beams. Each half of the split beam is captured at corresponding halves of the detector. FOV—field of view, kVp—peak kilovoltage, TR—time resolution.

Single-source split-filter DECT (Figure 8)

The TwinBeam DECT technology was commercialized by Siemens Healthineers. It has a single source and detector, with a split-filter of gold and tin at the x-ray tube, respectively, filtering the low- and high-energy beams. The spectrum is separated in two overlapped halves, each being captured at corresponding halves of the detector [2,5,6]. Recent scanners allow tube voltage selection of either 120 kV or 140 kV, the latter increasing spectral separation and tube output, which is useful in larger patients. Scanning can be performed at a FOV of 50 cm with a single fast gantry rotation of 0.28 s [35]. The pitch value is limited to 0.5 to maintain gapless imaging volume [6].

Dose reduction techniques are possible, enabling identical doses when compared with SECT [10,31]. Most importantly, it has lesser hardware complexity and lower cost, and may be incorporated as an upgrade to some scanner models [2,5].

Disadvantages include possible cross-scatter between the beams and the spectral overlap in the central 2–3 mm of the beam, precluding the use of that portion for material discrimination and limiting contrast [2]. There is also spectral mixture at the edges of the detector due to penumbra effects [15]. The low pitch results in greater scan time [12], which may compromise the degree of arterial enhancement in CT angiography [36]. Other drawbacks are the need for higher tube power to compensate for filtration that absorbs approximately two-thirds of the radiation, and the need for scanning each voxel at both energies, resulting in high delay and poor time resolution between the two energy scans. Additionally, the capability of balancing photon flux between low- and high-energy spectra is limited to filter capacity. Finally, the recent launch of the technology means there are fewer studies with this system [2,5].

**Figure 9:**
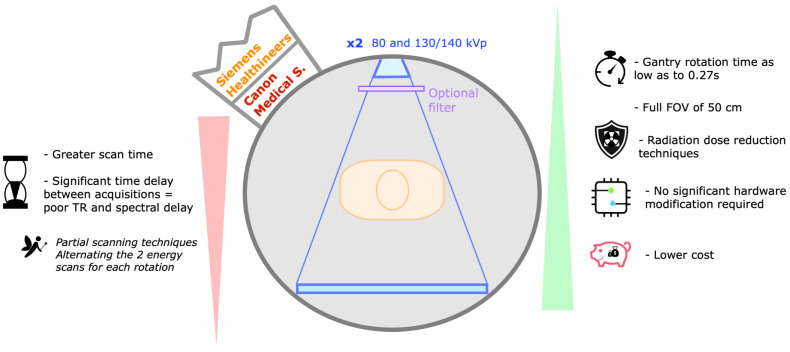
Single-source DECT with sequential acquisitions. Schematic representation of single-source DECT with sequential acquisitions and its drawbacks (listed on the left) and strengths (listed on the right). These scanners (commercialized by Siemens Healthineers and Canon Medical Systems) are composed of a single source and detector (optional filter). The low-energy scan typically uses 80 kVp, whereas the high-energy scan may use 130 or 140 kVp. The significant time delay between acquisitions leads to poor time resolution (TR) and spectral delay. FOV—field of view, kVp—peak kilovoltage, s—second.

Single-source DECT with sequential acquisitions (Figure 9)

Acquiring two consecutive scans at different energy levels is the most straightforward approach for acquiring DECT data, commercially available with two manufacturers (Siemens Healthineers and Canon Medical Systems). The low-energy scan typically uses 80 kVp, whereas the high-energy scan may use 130 or 140 kVp. An optional filter may be added. The entire FOV is scanned (50 cm) [5,6,10,35].

Its greatest advantage is that no significant hardware modification is required in the CT scanner. Additionally, dose reduction techniques can be used [2,5,35]. 

Disadvantages include the significant time delay between the high- and low-energy acquisitions, introducing temporal misregistration and limiting the evaluation of moving organs and changes in contrast opacification, so its clinical application is restricted to unenhanced studies [6,13]. For the same reason, patient motion between acquisitions can significantly distort spectral data. This delay may be minimized by alternating the two energy scans for each gantry rotation, instead of scanning the entire volume with multiple rotations at one energy followed by the other. Partial scanning techniques (obtaining projection data for only a part of the gantry rotation) may also improve temporal resolution for relatively static organs, although delays are still too long [1,5,15].

### 3.7. Is DECT Imaging Applicable to Daily Routine Workflow?

Dual-energy CT imaging has been associated with multiple workflow issues, including scheduling difficulties, increased reconstruction time, large number of images (increasing storage demands), and increased interpretation time [6,29].

Postprocessing DECT data has several implications, starting with access to software, which is exclusive and vendor-specific. Besides cost implications, it is also important to establish the availability of the desired capabilities that each can provide [12].

**Figure 10:**
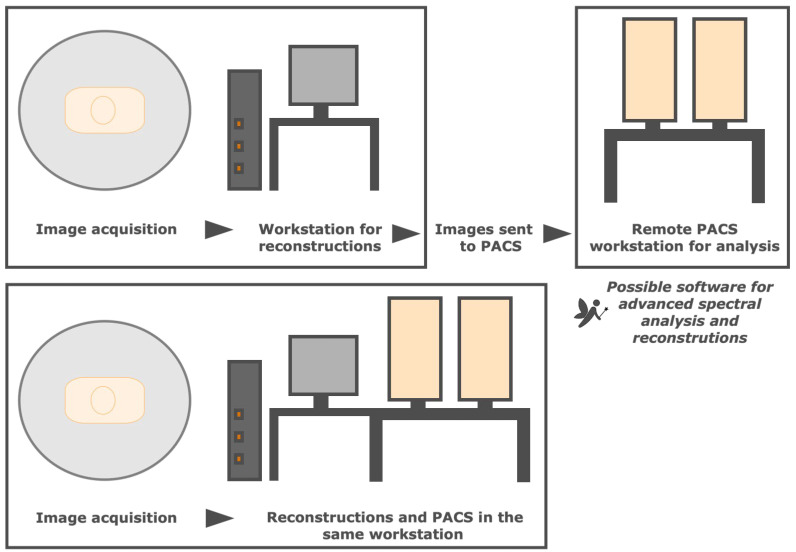
Different DECT workflow algorithms. Necessary reconstructions may be manually generated after the acquisition and the images sent to the picture archiving and communication system (PACS) for remote analysis at a different station (top representation), which may be complemented with advanced software that allows spectral CT analysis and reconstructions. alternatively, most vendors provide lighter workstation versions in the same computer as PACS (bottom representation), requiring a high level of vendor-specific scanner knowledge.

There are several workflow algorithms available (Figure 10). The necessary reconstructions may be manually generated after the acquisition and the images sent to the picture archiving and communication system (PACS). This facilitates the integration of DECT into routine clinical workflow, but the imaging technologist may spend more time per scan, and additional postprocessing is not possible if the full spectral dataset is unavailable [12,16]. Besides, the manipulation of some reconstructions in PACS may not always be optimal compared with an advanced workstation, although there is advanced software possible to integrate into the PACS of remote workstations that allow advanced spectral CT analysis, reconstructions, and the creation of decomposition maps [12,32]. Alternatively, most vendors provide lighter workstation versions in the same computer as PACS, but the user needs a high level of vendor-specific scanner knowledge, and a steep learning curve may be limitative [16].

### 3.8. What Are Beam Hardening Artifacts and How Are They Lessened?

Beam hardening artifacts originate from dense structures (e.g., bones) due to preferential attenuation of low-energy photons, increasing the mean photon energy of the detected x-ray spectrum and reducing the measured CT numbers in the reconstructed images [15,37]. Near-complete absorption of x-rays by very dense material leads to photon starvation. Beam hardening tends to provoke hypodense bands and streaks adjacent to dense objects, whereas photon starvation typically originates prominent streak artifacts [38]. These may degrade the representation of iodine distribution and lead to the inaccurate assessment of renal stones [15,28]. They can be reduced with image reconstruction in the projection domain [15], using 100 kVp for the low-energy acquisition, and high keV VMIs (although these also suppress iodine contrast) [28].

### 3.9. Is DECT Imaging Also Prone to Artifacts?

Yes, several artifacts may arise with DECT scans, some of which are inherent to the scanner design, acquisition protocol, and postprocessing techniques. In fact, some platforms exhibit unique artifacts that are not correctable through optimization of the scan parameters [15].

Regarding the reconstruction approach, when applied to the image domain image noise can increase, as previously described [5]. Optimizing image postprocessing parameters such as the reconstruction kernels and decomposition settings is essential to improve image quality in DECT examinations [15]. Reconstruction kernels are algorithms used to modify the frequency content of the image data prior to back projection, allowing image sharpening (higher spatial resolution, but increased image noise) or softening (low noise, but relatively low spatial resolution), depending on the specific objective of each image study. For instance, softer kernels are usually recommended for accurate attenuation measurements and sharper ones reduce the attenuation of atherosclerotic plaques [39].

Artifacts related to low photon counts (e.g., pixels mistakenly interpreted as enhancement or gouty deposition on material decomposition images) may be lessened by softer kernel selection, as well as by limiting the postprocessing to source data [15,40]. Pseudoenhancement can be reduced with VMIs (at ranges from 70 to 140 keV), due to the decrease in beam hardening artifacts [28,41]. Decomposition settings may be adjusted at the time of image interpretation to reduce artifacts, including the soft tissue density and decomposition ratio (between the two energy levels used to create the image), among others [40]. Notably, the decomposition ratio is influenced by spectral separation (increasing concordantly), and the optimized setting varies with the clinical purpose. Furthermore, incorrect attenuation thresholds for material decomposition algorithms may lead to false positive or negative results (e.g., tophus depiction). For virtual noncalcium image processing, the chosen suppression index must be adapted to the local calcium content, to avoid inadvertent suppression of bone [15].

Appropriate window and level settings are crucial for assessing the iodine content within structures and to avoid interpretation errors. Still, optimized iodine images are still prone to noise [28].

Artifacts related to patient size, temporal misregistration, and iodine concentration are discussed further.

### 3.10. Which DECT Scanners Are More Prone to Temporal Misregistration?

Consecutive scanning techniques are by far more prone to temporal misregistration and motion [1,2,5,15]. The orthogonal offset of the two source-detector pairs in dsDECT scanners induces spectral delay of about a quarter of the gantry rotation time, but its tube design largely improves the temporal resolution [15]. Split-filter DECT scanners require an entire spiral acquisition to ensure that each half of the split beam reaches the same imaging volume. Therefore, pitch values are low, resulting in increased breath hold time and relatively poor temporal registration [2,15,42].

**Figure 11:**
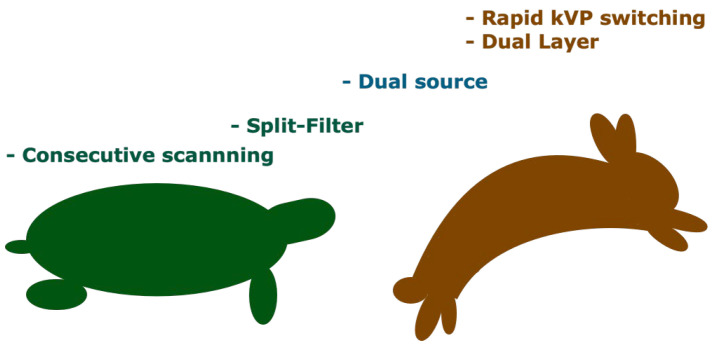
Variable temporal misregistration among DECT scanners. Consecutive scanning techniques are more prone to temporal misregistration and motion. Split-filter DECT scanners require an entire spiral acquisition, so the pitch values are low, resulting in relatively poor temporal registration. Although dual-source DECT scanners have an orthogonal offset of the 2 source-detector pairs inducing spectral delay, its tube design largely improves the temporal resolution. Temporal misregistration is negligible with rapid kVp switching and dual-layer DECT scanners.

Temporal misregistration is negligible with rapid kVp switching and dual-layer DECT scanners, given their inherent design (Figure 11) [15].

### 3.11. How Does the Patient Size Influence the DECT Image Quality?

**Figure 12:**
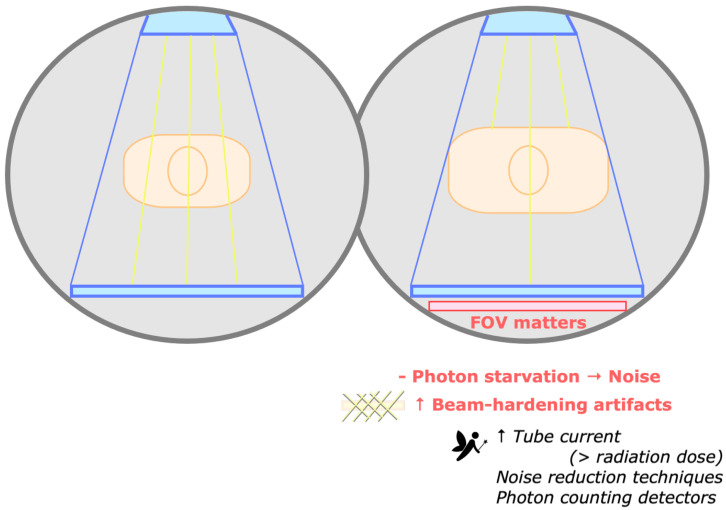
Impact of patient size in DECT image quality. Larger bodies may increase the image noise and reduce the image quality (given the insufficient number of photons reaching the detectors, represented by yellow lines). Beam hardening artifacts are more pronounced in larger patient sizes. Potential strategies to improve image quality include increasing the tube current and using noise reduction techniques. Noise is also reduced with the novel photon-counting scanners. The limited field of view (FOV) for dual-energy imaging in dual-source scanners may be a problem in larger patients.

Larger bodies may increase the image noise and reduce the image quality (given the insufficient number of photons reaching the detectors), degrading material decomposition and tissue characterization [15]. Additionally, beam hardening artifacts are more pronounced in larger patient sizes due to the greater path where x-rays are attenuated (Figure 12) [37]. As such, patient selection for DECT abdominal imaging has been recommended by using minimum cutoff values for weight and transverse dimension based on the frontal scout radiograph [28,43].

As photon starvation is more marked at low kVp, this problem is more apparent with DECT scanners restricted to a low-energy option of 80 kVp (e.g., rapid kVp switching and some dsDECT scanners) [14,28]. Image quality may be improved by increasing the tube current, slowing the rotation time, and reducing the pitch, all of which increase the photon flux, at the cost of higher radiation dose (lessened with tube current modulation). The limited FOV for dual-energy imaging in dsDECT scanners (26–35.5 cm) may be a problem in larger patients, although they may be positioned so that the area/organ of interest lies within the spectral FOV [15].

Photon-counting detector CT scanners are promising for imaging large patients, reducing electronic noise, and improving CT number stability at low photon flux [44,45]. For now, noise-reduction iterative reconstruction techniques may be used to improve image quality in large patients [15].

### 3.12. How Is Contrast Enhancement Improved with DECT and Why Is It Advantageous?

**Figure 13:**
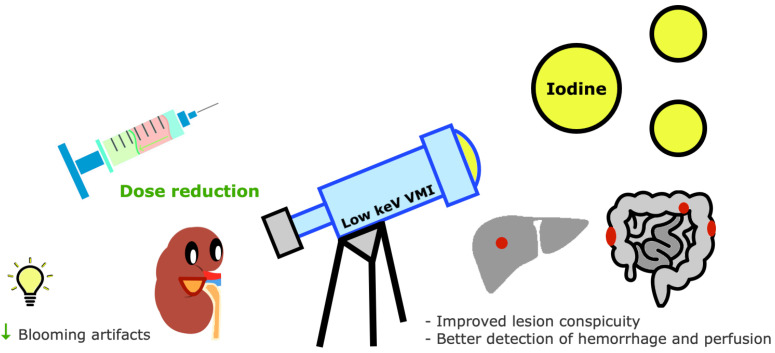
Contrast enhancement improvement with DECT imaging. Iodine density is amplified on low-keV virtual monochromatic images (VMIs). The improved sensitivity for iodine with DECT allows a reduction of iodine dose, which is useful in patients with compromised renal function and reduces blooming artifacts. Other advantages include improved lesion conspicuity and better detection of hemorrhage and perfusion assessment.

Given its k-edge of 33.2 keV, iodine density is amplified on low-keV VMIs [15]. An important advantage of the improved sensitivity for iodine with DECT is the potential reduction of the iodine dose, which is useful in patients with impaired renal function (Figure 13) [29,46].

Improved contrast of iodine maps makes enhancing lesions or vessels more conspicuous and allows better delineation of bowel ischemia and lung hypoperfusion [6,47].

On the other hand, iodine may originate blooming artifacts, especially with oral contrast medium, but these are potentially mitigated by changing window settings and reducing iodine concentration, which also improves the quality of VNC images [15].

Studies comparing iodine sensitivity among DECT scanners concluded that it correlates mainly with better spectral performance and is also influenced by other factors such as image reconstruction techniques, patient size, and lesion location [34,47,48,49,50].

### 3.13. Is DECT Imaging Associated with Greater Radiation Exposure?

Although radiation dose was an initial concern with DECT, it is no longer an issue due to technological improvements and refinements [12]. In fact, several studies found similar or lower radiation doses compared to SECT, with no significant differences in image quality [4,8,29,51,52]. Still, radiation dose may differ significantly among scanners, and varies with the type of scan, body part, and patient-specific factors [12].

Strategies for dose reduction include the use of VNC images, noise-reduction iterative reconstruction algorithms, and limiting the FOV to the area of interest. Scanner-specific strategies include attributing relatively more time in the low kVp energy in fast kVp switching DECT, modulating the tube current and applying a filter to the higher-energy tube in dsDECT [46,53], and removing low-energy photons in split-filter DECT [14].

Spectral contrast is also important when comparing radiation doses between DECT scanners, since better spectral separation allows a certain image quality with less dose [54]. It is maximally achieved with dsDECT systems with optimized voltage, current, and filtration [13].

Regarding ultralow-dose protocols such as pediatrics and CT colonography, currently, DECT has a minimal or absent role, except for its potential to eventually allow minimal preparation CT colonography with subtraction of contrast-tagged fecal material [46,53].

### 3.14. How Do DECT Systems Differ in Terms of Imaging Quality?

Current commercially available DECT platforms have technological differences that may influence the spectral performance and, consequently, lesion quantification and characterization [55]. Some scanners have proven superiority in image quality over the others (dual-source, fast kV-switching, and dual-layer), possibly due to variable spectral overlap and reconstruction algorithms [34,55,56]. These require computing systems of significantly high performance and are less cost-effective for routine practice. The simpler and cheaper approach of sequential scanning using two energy levels comes with the tradeoff of poor temporal resolution. Split-filter DECT technique is available on medium-performance, more affordable systems [56]. Comparative studies need to be renewed as the DECT platforms keep being upgraded.

### 3.15. Are DECT Images Reproducible among Different Scanners?

Reproducibility is particularly important in routine oncological imaging. Unfortunately, the variability in material-specific decomposition methods among manufacturers remains limitative. In fact, several studies reported marked inter-vendor variability in terms of monochromatic data and iodine quantification, so studies using different scanners and different VMI energy should be interpreted with caution [16,57].

### 3.16. What Are the Main Clinical Applications of DECT?

DECT provides several advantages in clinical practice, including the potential for better lesion depiction and characterization (related to CNR); radiation dose reduction by generating VNC images that eliminate the need for a non-contrast phase; differentiating between hemorrhage, enhancement, or calcification; and reducing metal-related artifacts, among others [7,29]. The accurate characterization of incidental findings may obviate the need for further imaging, reducing the costs, radiation dose, and patient anxiety [58,59]. Its benefits are also well-known in radiation oncology, namely the improved dose calculation, disease visibility, and personalized treatment, being particularly useful for dose delivery techniques with a steeper dose gradient, such as brachytherapy and proton therapy. It provides more quantitative measurements compared with SECT and is less prone to image noise and beam hardening or metal artifacts, improving the accuracy of the calculated dose [60,61,62]. 

**Figure 14:**
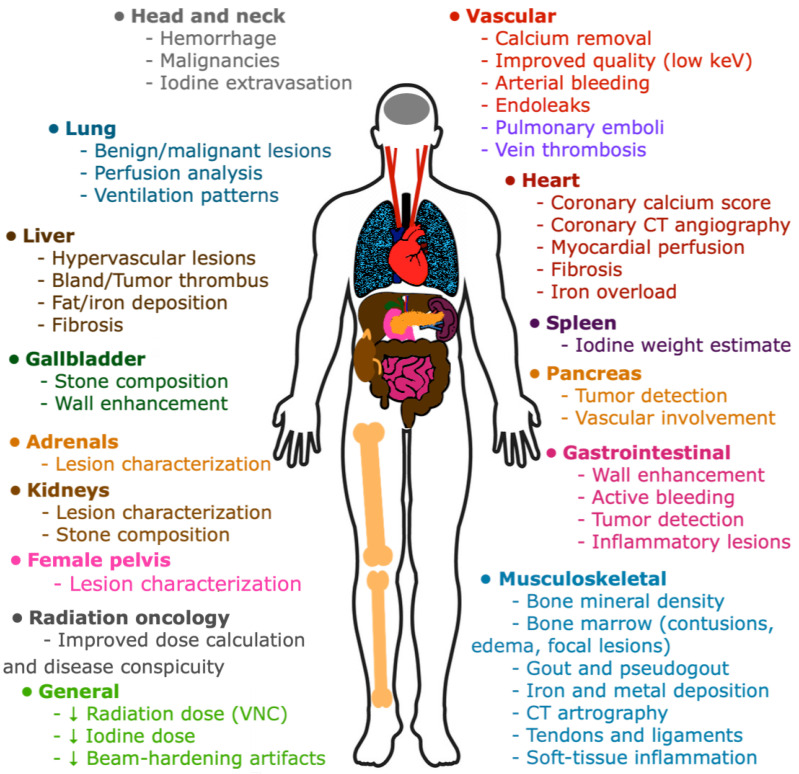
Main clinical applications of DECT. Dual-energy CT provides several advantages, including the potential for better lesion depiction and characterization, radiation dose reduction by generating virtual non-contrast (VNC) images, iodine dose reduction, and beam-hardening artifacts reduction. Its benefits are also well-known in radiation oncology. Examples of the many useful applications that have been reported across all body systems are listed here.

The main clinical applications of DECT are listed in Figure 14 [4,14,16,17,20,29,38,46,63,64,65,66,67,68,69,70,71].

### 3.17. What to Expect in the Future of Spectral Imaging?

Photon-counting scanners are advanced spectral CT systems that have been under investigation and development for more than ten years. The first clinical CT system using this technology was launched in 2021 by Siemens Healthineers [45].

These scanners have detectors with cadmium-based semiconductors that split a single x-ray energy spectrum into more than two photon energy bins, enabling new approaches for material decomposition, such as K-edge imaging and classification of materials at very low concentrations [1,5,6,7]. They can reduce the electronic circuit noise, beam hardening, and metal artifacts; increase iodine CNR, spectral separation, and spatial resolution; and generate greater quality VMIs. Additionally, dose efficiency is improved [1,45,72,73,74]. Labeling nanoparticles with elements that may be detected by K-edge imaging allows for molecular CT imaging, and the injection of multiple contrast agents (such as iodine and gadolinium) allow multiple-phase images at the same time, reducing the number of acquisitions and radiation doses, although further research is needed before human scanning approval [7,45,75].

Its application on CT was delayed due to significant technical restraints caused by high exposure rates and the photon flux required for CT [1,5]. Some physical challenges remain: For instance, there is substantial overlap between the different energy bins (possibly corrected) and the system must be capable of ultrafast photon processing (possible due to new developments). If the signal process is slow, an overlap of electric pulses occurs (“pulse pile-up”) and two or more photons are detected as one higher-energy photon, underestimating the count (possibly lessened by several strategies) [6,7,45].

Still, further improvement of photon-counting detector CT imaging is expected, for which iterative reconstruction and deep learning techniques may be useful [45]. 

Other investigated methods to expand multi-energy evaluation capabilities of spectral CT include combining dual-source and TwinBeam technologies, using multi-kVp imaging, and switching the x-ray tube between multiple voltages [5].

## 4. Conclusions

The added value of DECT has been widely validated in many settings. Material decomposition techniques and improved image quality expand the diagnostic capability and accuracy among many disease processes. Furthermore, the possibility of reducing the contrast agent and radiation dose is valuable in the current days of widespread imaging. In a world full of diversity, it is of extreme relevance to acknowledge the strengths and weaknesses of different DECT scanners, which can be adapted to each specific context, in terms of the patient, clinical question, and economic capacity. Although “one scanner does not fit all” clinical, demographic, and financial scenarios, DECT technology is undoubtedly valuable in the clinical practice and will certainly keep improving.

## Figures and Tables

**Table 1:** List of studies included in the review (reference number), with indication of the topics of analysis for the current review (X).

Topic of Analysis Article	DECT Technique *	Scanner Specifications **	Clinical Applications	Future Perspectives
[1]	X	X		X
[2]	X	X		
[3]	X			
[4]	X		X	
[5,6]	X	X		X
[7]	X		X	X
[8,9]	X			
[10]	X	X		
[11]	X			
[12,13]	X	X		
[14]	X	X	X	
[15]	X	X		
[16]	X	X	X	
[17]	X		X	
[18,19]	X			
[20]	X		X	
[21,22,23,24,25,26,27,28]	X			
[29]	X		X	
[30]	X			
[31]		X		
[32]	X	X		
[33]		X		
[34]	X	X		
[35,36]		X		
[37]	X			
[38]	X		X	
[39,40,41,42,43]	X			
[44,45]	X			X
[46]	X		X	
[47,48,49,50]	X	X		
[51,52,53,54]	X			
[55,56,57]	X	X		
[58,59,60,61,62,63,64,65,66,67,68,69,70,71]			X	
[72,73,74]				X

* Physical concepts, image quality, radiation considerations, postprocessing methods, advantages, and limitations. ** Design, image quality, advantages, limitations, interscanner reproducibility.

## Data Availability

Not applicable.

## Abbreviations

CNR	contrast-to-noise ratio
CT	computed tomography
DECT	dual-energy computed tomography
dsDECT	dual-source dual-energy computed tomography
FOV	field of view
keV	kiloelectronvolt
kVp	kilovolt peak
VMIs	virtual monochromatic images
VNC	virtual non-contrast
PACS	picture archiving and communication system
SECT	single-energy computed tomography
